# Escitalopram Ameliorates Cardiomyopathy in Type 2 Diabetic Rats via Modulation of Receptor for Advanced Glycation End Products and Its Downstream Signaling Cascades

**DOI:** 10.3389/fphar.2020.579206

**Published:** 2020-12-15

**Authors:** Lamiaa A. Ahmed, Nesma A. Shiha, Amina S. Attia

**Affiliations:** Department of Pharmacology and Toxicology, Faculty of Pharmacy, Cairo University, Cairo, Egypt

**Keywords:** cardiomyopathy, depression, diabetes mellitus, escitalopram, metabolic derangements 3

## Abstract

Type 2 diabetes mellitus (T2DM) has been recognized as a known risk factor for cardiovascular diseases. Additionally, studies have shown the prevalence of depression among people with diabetes. Thus, the current study aimed to investigate the possible beneficial effects of escitalopram, a selective serotonin reuptake inhibitor, on metabolic changes and cardiac complications in type 2 diabetic rats. Diabetes was induced by feeding the rats high fat-high fructose diet (HFFD) for 8 weeks followed by a subdiabetogenic dose of streptozotocin (STZ) (35 mg/kg, i. p.). Treatment with escitalopram (10 mg/kg/day; p. o.) was then initiated for 4 weeks. At the end of the experiment, electrocardiography was performed and blood samples were collected for determination of glycemic and lipid profiles. Animals were then euthanized and heart samples were collected for biochemical and histopathological examinations. Escitalopram alleviated the HFFD/STZ-induced metabolic and cardiac derangements as evident by improvement of oxidative stress, inflammatory, fibrogenic and apoptotic markers in addition to hypertrophy and impaired conduction. These results could be secondary to its beneficial effects on the glycemic control and hence the reduction of receptor for advanced glycation end products content as revealed in the present study. In conclusion, escitalopram could be considered a favorable antidepressant medication in diabetic patients as it seems to positively impact the glycemic control in diabetes in addition to prevention of its associated cardiovascular complications.

**GRAPHICAL ABSTRACT:**
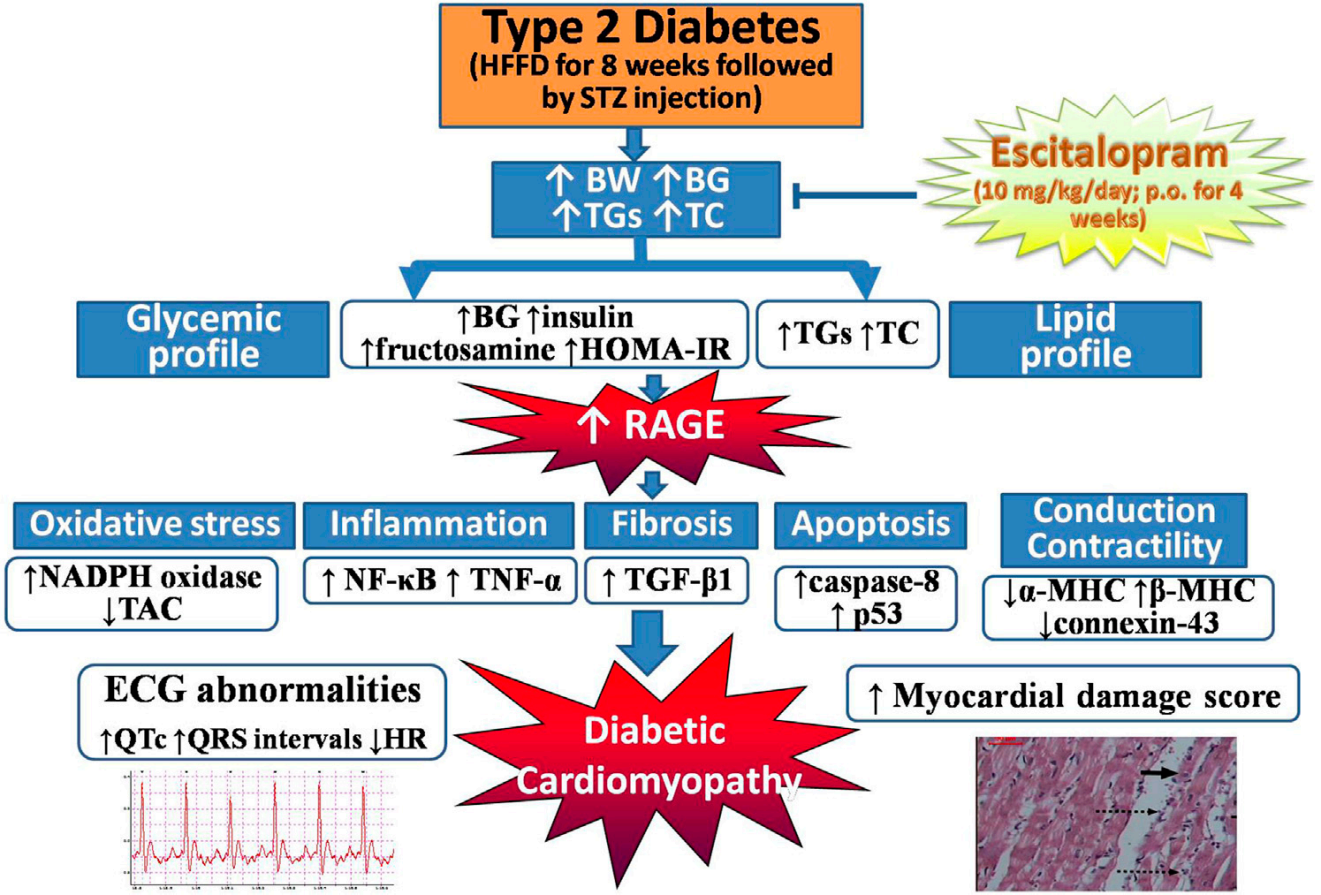
Escitalopram alleviates HFFD/STZ-induced metabolic and cardiac derangements in type 2 diabetic rats. BG: blood glucose, BW: body weight, ECG: electrocardiographic, HFFD: high fat-high fructose diet, HOMA-IR: homeostasis model assessment of insulin resistance, HR: heart rate, MHC: myosin heavy chain, NADPH oxidase: nicotinamide adenine dinucleotide phosphate oxidase, NF-κB: nuclear factor-kappa B, RAGE: receptor for advanced glycation end products, STZ: streptozotocin, TAC: total antioxidant capacity, TC: total cholesterol, TGF-β: transforming growth factor-beta, TGs: triglycerides, TNF-α: tumor necrosis factor-alpha.

## Introduction

Type 2 diabetes mellitus (T2DM) has been recognized as a known risk factor for cardiovascular diseases affecting approximately 32.2% of all persons with T2DM (Einarson et al., 2018). Both clinical and experimental studies have highlighted the existence of a specific diabetic cardiomyopathy (DCM) (Davidoff et al., 2004; De Rosa et al., 2018) which is attributed to structural and functional changes of the myocardium, independent of other coexisting heart conditions (Miki et al., 2013; Gulsin et al., 2019).

The exact pathophysiology of DCM is multifactorial where advanced glycation end products (AGEs) formation and accumulation secondary to chronic hyperglycemia has been established as an important contributor to the development of DCM (Bodiga et al., 2014; Athithan et al., 2019). Stimulation of receptor for advanced glycation end products (RAGE) by AGEs activates nicotinamide adenine dinucleotide phosphate (NADPH) oxidase, enhancing the generation of reactive oxygen species (ROS) which in turn play a pivotal role in the diabetes-induced cardiovascular damage (Jay et al., 2006).

Furthermore, the stimulated RAGE induces the activation and the translocation of the oxidative stress related transcription factor; nuclear factor-kappa B (NF-κB) (Luevano-Contreras and Chapman-Novakofski, 2010). NF-κB is a central coordinator of the pro-inflammatory genes expression, controlling the expression of pro-inflammatory cytokines (tumor necrosis factor-alpha (TNF-α), interleukin (IL)-1β, IL-6) and adhesion molecules (vascular cell adhesion molecule-1). In addition, sustained TNF-α signaling activation induces cardiomyocyte apoptosis through the activation of both intrinsic and extrinsic cell death pathways (Haudek et al., 2007). Pro-inflammatory cytokines can also affect cardiac contractility where they exert negative inotropic effects on the heart, resulting in a rapid contractile dysfunction and interfering with the excitation-contraction coupling (Frati et al., 2017).

Additionally, interstitial fibrosis is a structural hallmark of DCM (Mizamtsidi et al., 2016). Transforming growth factor-beta (TGF-β) induces fibroblasts differentiation to myofibroblasts with production of much collagen (Petrov et al., 2002). Collagen can undergo glycation by AGEs, resulting in cross-linking and impairing its degradation, leading to fibrosis and myocardial stiffness (Wang et al., 2006).

Experimental studies revealed an association between depressive like behaviors and T2DM (Ye et al., 2017; Soliman et al., 2020). Clinically, depression has been found to negatively impact glycemic control and to increase the diabetic complications including cardiovascular diseases (Lin et al., 2010; Oladeji and Gureje, 2013). Growing body of evidence also suggests a bidirectional association between diabetes and depression in patients with each disease increasing the risk of the other (Zhuang et al., 2017). Importantly, there have been concerns about adverse effects of some antidepressants (e.g., tricyclic antidepressants and monoamine oxidase inhibitors) on glucose metabolism, at least in part through inducing significant weight gain and insulin resistance (Goodnick, 2001; Barnard et al., 2013; Gehlawat et al., 2013). The use of some antidepressants also induces serious cardiovascular complications including arrhythmia and sudden cardiac death (Leonard et al., 2011). Hence, the need for an antidepressant with no adverse effects on the metabolic features of diabetes and its cardiac complications is warranted.

Selective serotonin reuptake inhibitors (SSRIs) are commonly prescribed antidepressants due to their favorable safety profile and efficacy (Markowitz et al., 2011). Escitalopram is a SSRI which is used as a first-line option in the management of major depression and anxiety disorders (Kirino, 2012). In addition to its antidepressant effect, escitalopram has demonstrated antioxidant, anti-inflammatory and antihyperlipidemic effects, both experimentally and clinically (Eren et al., 2007; Unis et al., 2014; Arain et al., 2017; Abdo et al., 2019). Moreover, the use of escitalopram in diabetic and depressed patients was associated with a possible beneficial effect on glycemic control without inducing weight gain (Gehlawat et al., 2013). Escitalopram also exhibits distinct advantages in comparison to other SSRIs regarding its cardiovascular safety (Guo et al., 2019).

Therefore, the current study was designed to investigate if escitalopram, a potent and a well-known antidepressant, would have favorable or adverse effects on metabolic changes and cardiac complications associated with T2DM in rats.

## Material and Methods

### Animals

Adult male Wistar albino rats, weighing 80–120 g, were obtained from the National Research Center Laboratory, Cairo, Egypt. Rats were housed in standard polypropylene cages in the animal house of Faculty of Pharmacy, Cairo University under constant environmental conditions and a 12 h light-dark cycle. Animals were allowed to acclimate for at least 7 days prior to dietary manipulation and were fed normal pellet diet and tap water *ad libitum*. All experimental procedures were approved by the Ethics Committee, Faculty of Pharmacy, Cairo University (Permit Number: PT 1303) and were conducted in compliance with the *Guide for Care and Use of Laboratory Animals* published by the US National Institutes of Health (NIH Publication No. 85-23, revised 2011).

### Drugs and Chemicals

Streptozotocin (STZ) was purchased from Sigma-Aldrich, USA. Fructose was obtained from El-Nasr Pharmaceutical chemicals Company, Egypt and the long-acting insulin (Insulatard) from Novo Nordisk A/S, Denmark. Escitalopram oxalate (Cipralex) was purchased from H. Lundbeck A/S, Denmark. Escitalopram was freshly prepared in saline immediately before use and administered orally. All other chemicals and reagents, unless specified, were obtained from Sigma-Aldrich, United States.

### Experimental Design

Fifty rats were divided into two dietary regimen-groups; normal fat diet (NFD, n = 20) and high fat-high fructose diet (HFFD, n = 30) by combining an in-house-prepared HFD with fructose in drinking water (20%) for a period of 8 weeks. HFD provided 5.3 kcal/g and composed of fat (15%:14% saturated animal fat and 1% cholesterol powder), protein (21%), carbohydrate (60%), fibers (3%), vitamins and minerals (1%). During the 8^th^ week, HFFD group received a single daily dose of insulin (0.5 IU/kg, i. p.) to enhance the development of insulin resistance (IR) (Chang et al., 1999) and to guard against the decrease in insulin level following STZ injection (Srinivasan et al., 2005; Schaalan et al., 2009). At the beginning of the 9^th^ week, a single subdiabetogenic dose of STZ (35 mg/kg) freshly prepared in citrate buffer (0.1 M, pH 4.5) was injected i. p. into each rat after an overnight fasting to produce frank hyperglycemia (Schaalan et al., 2009). The NFD rats received an equivalent volume of citrate buffer. In order to protect the HFFD/STZ rats from STZ-induced hypoglycemia, they were given 5% oral glucose solution in drinking water during the first 24 h after STZ administration (Hajduch et al., 1998). HFFD regimen was then stopped and animals were fed normal diet for the rest of the study. 1 week after STZ injection, rats with persistent blood glucose levels between 200 and 350 mg/dL, hyperinsulinemia and dyslipidemia were considered insulin resistant/T2DM and were selected for further pharmacological studies.

#### Intraperitoneal Glucose Tolerance Test

Intraperitoneal glucose tolerance test (IPGTT) was performed 1 week after STZ administration. Six-hour fasted diabetic and non diabetic rats were administered i. p. dose of glucose (2 g/kg) (Elmazar et al., 2013). Blood droplets were withdrawn from the tail vein at zero time and every 30 min along 2 h to estimate the resulting blood glucose levels and to confirm the IR state. Glucose was measured using Accu-Check Active glucometer (Roche Diagnostics, Germany). Area under the curve (AUC) was calculated according to the following equation (Psyrogiannis et al., 2003):

AUC = 0.25 (fasting value) + 0.5 (1/2 h value) + 0.75 (1 h value) + 0.5 (2 h value).

#### Main Experimental Groups

Half of NFD fed rats (n = 10) received saline and served as normal group. The other half (n = 10) received escitalopram (10 mg/kg/day, p. o.) (Eren et al., 2007; Unis et al., 2014) and served as escitalopram group. Diabetic rats that fulfilled the aforementioned criteria were randomly assigned into two other groups, each containing 10 rats. One group served as HFFD/STZ diabetic rats and received saline while the last group was orally treated with escitalopram (10 mg/kg/day). Treatment was continued for further 4 weeks till the end of experiment.

### Blood and Tissues Sampling

**FIGURE 1:**
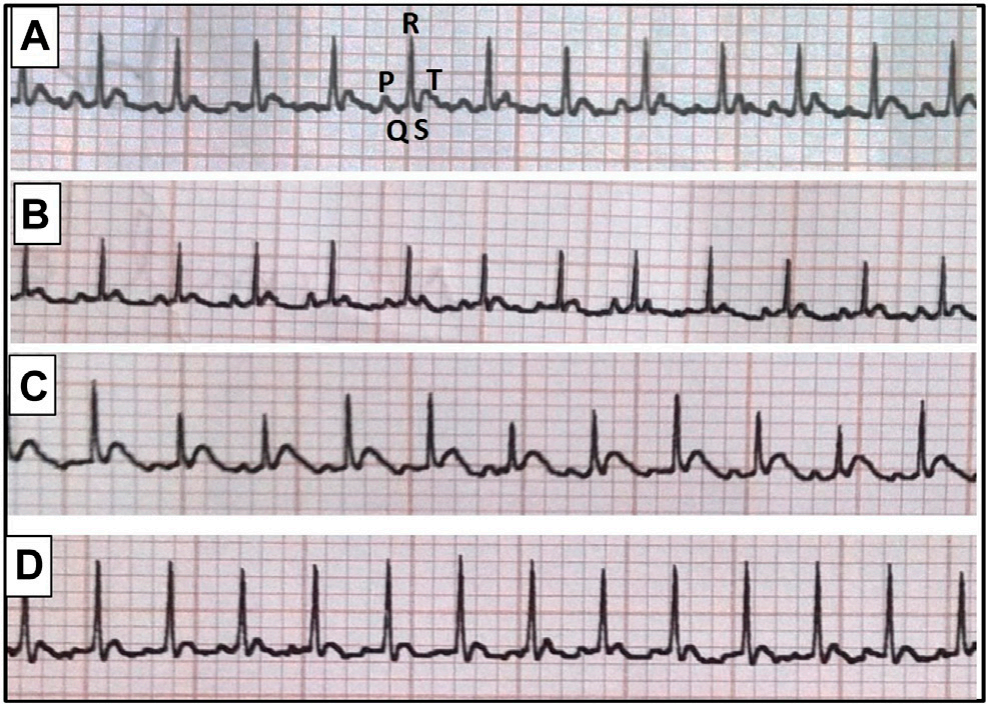
Representative electrocardiographic images for normal group **(A)**, escitalopram group **(B)**, diabetes group **(C)** and diabetes + escitalopram group **(D)**.

At the end of the experimental period, rats were anesthetized with thiopental (50 mg/kg, i. p.) and kept warmed to prevent the incidence of hypothermia. Subcutaneous peripheral limb electrodes were inserted for electrocardiographic (ECG) recording (HPM 7100, Fukuda Denshi, Tokyo, Japan) to determine heart rate (HR) as well as QT interval and QRS duration (Figure 1). Animals were then weighed and blood samples were collected under anesthesia from the retro-orbital sinus into non-heparinized tubes. Rats were fasted for 12 h before blood sampling in order to minimize feeding-induced variations in glucose and lipid patterns. Separated serum samples were stored at −20°C for estimation of glucose, insulin, fructosamine, triglycerides (TGs) and total cholesterol (TC) levels.

After the collection of blood samples, animals were euthanized by cervical dislocation. The whole ventricles were rapidly excised, washed with ice-cold saline then weighed after blotting with filter paper. For each group, two sets of experiments were conducted; one for electrocardiographic and biochemical measurements (n = 6) and the other (n = 4) for histological examination. For biochemical measurements, the whole ventricle was divided into three transverse parts. One part was homogenized in ice cold saline using a homogenizer (Heidolph Diax 900, Germany) to prepare 10% w/v homogenate and the resultant homogenates were centrifuged at 5000 rpm for 10 min at 4°C using cooling centrifuge (Hettich universal 32A, Germany). The separated supernatants were stored at −80°C till the required measurements where protein contents were estimated as demonstrated by Lowry et al*.* (1951). The other two parts of ventricles were used for real time-PCR and western blot analyses.

### Biochemical Measurements

#### Determination of Glycemic and Lipid Profiles

Serum glucose, fructosamine and insulin levels were determined using enzymatic colorimetric kit (Biodiagnostic, Egypt, Cat.# GL 13 20), rat fructosamine ELISA kit (Nova lifetech Science, Hong Kong) and rat insulin ELISA kit (Abnova, Taiwan, Cat.# KA3811), respectively. The kits procedures were performed in line with the manufacturer's instructions then expressed as mg/dL, μmol/L and μIU/mL, respectively. Homeostasis model assessment of insulin resistance (HOMA-IR) was calculated according to the equation demonstrated by Matthews et al. (1985). On the other hand, serum TGs and TC levels were determined using enzymatic colorimetric kits (Spinreact, Spain) according to the manufacturer's instructions and the results were expressed as mg/dL.

#### Determination of Myocardial Receptor for Advanced Glycation End Products and Oxidative Stress Biomarkers

The estimation of myocardial contents of RAGE and NADPH oxidase two was carried out using commercially available rat ELISA kits (RayBiotech Inc., United States, Cat.# ELR-RAGE and Bioassay Technology Laboratory, China, Cat.# E1173Ra, respectively), while total antioxidant capacity (TAC) was estimated using ABTS antioxidant assay kit (ZenBio Inc., USA). The procedures of the used kits were performed along with the manufacturer's instructions, where the results were expressed as ng/mg protein for RAGE and NADPH oxidase two and μM/mg protein for TAC.

#### Determination of Myocardial Inflammatory and Fibrogenic Biomarkers

The inflammatory (NF-κB p65 and TNF-α) as well as fibrogenic (TGF-β1) markers were estimated using rat ELISA kits from Elabscience (China), Cat.# E-EL-R0674; RayBiotech Inc. (USA), Cat.# ELR-TNFa and Kamiya Biomedical Company (USA), Cat.# KT-30309, respectively and the results were expressed as ng/mg protein for NF-κB p65 and pg/mg protein for the other 2 markers.

#### Determination of Myocardial Apoptotic Biomarkers

Myocardial contents of caspase-8 (the initiator caspase of the extrinsic apoptotic pathway) as well as P53 (the stimulator of intrinsic apoptotic pathway) were assessed using rat caspase-8 ELISA kit (Cusabio, China, Cat.# CSB-E14912r-24) and p53 pan ELISA kit (Roche Diagnostics, Germany, Cat.# 11 828 789 001) and the results were expressed as ng/mg protein and pg/mg protein, respectively. Furthermore, caspase-3 activity (the key enzyme in the execution of both extrinsic and intrinsic apoptosis) was estimated using caspase-3/CPP32 colorimetric assay kit (Biovision Milpitas, CA, USA, Cat.# K106-100) where the results were expressed as nmol pNA/h/mg protein.

### Detection of α-and β-Myosin Heavy Chain Gene Expression

Part of the ventricle was used to assess myocardial α- and β-myosin heavy chain (MHC) gene expression using quantitative real time-PCR technique. In brief, total RNA was extracted from heart tissues using SV total RNA isolation system (Promega, USA), and the purity of the obtained RNA was verified spectrophotometrically at 260/280 nm. The extracted RNA was then reverse transcribed into complementary DNA using Reverse Transcription System (Promega, USA). Quantitative real time-PCR was performed using SYBR Green JumpStart Taq ReadyMix (Sigma-Aldrich, USA) as described by the manufacturer; sequences of the primers used are listed in Table 1. After the quantitative real time-PCR run, the relative expression of target genes was obtained using the 2^−∆∆CT^ formula with beta-actin (β-actin) as a housekeeping gene (Livak and Schmittgen, 2001).

**TABLE 1:** Sequences of the primers used.

Genes	Forward primers	Reverse primers
α-MHC	5′-GACACCAGCGCCCACCTG-3′	5′-ATAGCAACAGCGAGGCTCTTTCTG-3′
β-MHC	5′-GGAGCTCACCTACCAGACAGA-3′	5′-CTCAGGGCTTCACAGGCATCC-3′
β-Actin	5′-TATCCTGGCCTCACTGTCCA-3	5′-AACGCAGCTCAGTAACAGTC-3

### Western Blot Analysis of Connexin-43

Another part of the ventricle was homogenized in lysis buffer and quantified for protein levels using a Bicinchoninic acid protein assay (BCA) kit (Thermo Fisher Scientific Inc., USA). Protein expression was assessed as previously described (Ahmed et al., 2014) using connexin 43/GJA1 primary antibody (R&D Systems Inc., USA, Cat.# PPS045) and horseradish peroxidase (HRP)-conjugated goat anti-mouse secondary antibody (Novus Biologicals, USA, Cat.# HAF007). The amount of protein was assessed by densitometric analysis of the autoradiograms using a scanning laser densitometer (Biomed Instrument Inc., USA). Results were expressed as arbitrary units after normalization for β-actin protein expression.

### Histological Assessment of Myocardial Damage

The whole ventricle was rinsed in ice-cold saline, then portions were collected from different areas (the base, middle, and apex) and immediately fixed in 10% formalin for 24 h. The specimens were then washed, dehydrated in ascending grades of ethanol, cleared in xylene and embedded in paraffin wax. Serial sections (5 μm thick) were obtained, stained with haematoxylin and eosin (H&E) and examined microscopically (magnification x200) using an image analyzer (Leica Qwin 550, Germany). Myocardial damage was evaluated as demonstrated by Zhang et al. (2008) using a semi-quantitative grading scale of 0–5 (0, normal myocardial cells; 1, <5% of myocardial cells showing necrosis with one or two apoptotic cells/field; 2, 5–15% of myocardial cells showing necrosis with no more than four apoptotic cells/field; 3, 16–25% of myocardial cells showing necrosis with up to five apoptotic cells/field; 4, 26–35% of myocardial cells showing necrosis in confluent areas with up to six apoptotic cells/field and 5, >35% of myocardial cells showing necrosis in multiple massive or coalescent areas with up to seven or more apoptotic cells/field). Histological changes were evaluated by a pathologist unaware of different groups examined.

### Statistical Analysis

Data were expressed as mean ± standard error of mean (SEM). Results were analyzed using one way analysis of variance test (One-way ANOVA) followed by Tukey’s multiple comparisons test except the results of IPGTT which was done using two-way repeated measures ANOVA followed by Bonferroni's multiple comparisons test and its AUC was done using unpaired Student’s t test. Additionally, histological score of damage was done using non-parametric One-Way ANOVA followed by Dunn's multiple comparison test. Statistical analysis was performed using GraphPad Prism software, version 6 (GraphPad Software Inc., United States). For all the statistical tests, the level of significance was fixed at *p* < 0.05.

## Results

Overall, no significant differences were observed between the assessed parameters in normal rats treated with escitalopram compared with those of untreated normal rats.

### Changes in Body Weight as Well as Serum Glucose, Triglycerides and Total Cholesterol Levels During Induction of HFFD/STZ Type 2 Diabetes in Rats

Feeding rats with HFFD for 8 weeks accompanied by a single daily dose of insulin (0.5 IU/kg, i. p.) during the 8^th^ week resulted in a significant increase in their body weight (BW) as well as serum glucose, TGs and TC levels by about 165%, 52%, 184% and 79%, respectively compared to their initial values. Moreover, administration of STZ (35 mg/kg, i. p.) at the beginning of the 9^th^ week caused further increase in serum glucose level by about 156% compared to that at the end of the 8^th^ week (Table 2).

**TABLE 2:** Changes in body weight as well as serum glucose, triglycerides and total cholesterol levels during induction of HFFD/STZ type 2 diabetes in rats.

Time	BW (g)	Serum glucose (mg/dL)	Serum TGs (mg/dL)	Serum TC (mg/dL)
Start (week 0)	98.00 ± 4.09	81.40 ± 1.80	46.38 ± 2.38	53.37 ± 0.65
8^th^ week (HFFD + insulin)	260.00 ± 6.79*	123.60 ± 2.69*	131.80 ± 7.11*	95.27 ± 5.24*
1 week after STZ	252.30 ± 2.13*	316.20 ± 14.60*^#^	131.20 ± 8.97*	87.35 ± 5.49*

BW: body weight, HFFD: high fat-high fructose diet, STZ: streptozotocin, TC: total cholesterol, TGs: triglycerides. Each value represents the mean of six experiments ±SEM. Statistical analysis was done using One way ANOVA followed by Tukey’s post-hoc test. *p < 0.05 vs. week 0, ^#^ *p* < 0.05 vs. 8^th^ week.

### Intraperitoneal Glucose Tolerance Test and Area Under the Curve in Normal and HFFD/STZ Type 2 Diabetic Rats

One week following STZ injection, the HFFD/STZ model group showed impaired glucose tolerance upon glucose administration (2 g/kg, i. p.), where blood glucose levels in the HFFD/STZ diabetic group were much higher than that in the normal group at 0, 30, 60, 90 and 120 min by about 204, 134, 266, 255, and 227%, respectively. This was also confirmed by the calculated AUC which was significantly higher in the HFFD/STZ diabetic group than the normal group by 206.47% (Table 3).

**TABLE 3:** Intraperitoneal glucose tolerance test and area under the curve in normal and HFFD/STZ type 2 diabetic rats.

Groups	Blood glucose level (mg/dL)					AUC (mg/dL)
	0 min	30 min	60 min	90 min	120 min	
Normal	96.15 ± 4.29	160.30 ± 13.80	108.80 ± 4.80	101.40 ± 7.37	98.85 ± 4.73	235.20 ± 13.86
Diabetes	292.30 ± 4.95*	375.20 ± 17.87*	398.30 ± 23.14*	360.20 ± 22.41*	322.90 ± 25.14*	720.81 ± 37.35*

AUC: area under the curve. Each value represents the mean of six experiments ±SEM. Statistical analysis was done using two-way repeated measures ANOVA followed by Bonferroni's multiple comparisons test except for AUC which was done using unpaired Student’s t test. *p < 0.05 vs. normal.

### Effect of Escitalopram on Body Weight and Heart Weight Index as Well as Electrocardiographic Changes in HFFD/STZ Type 2 Diabetic Rats

The HFFD/STZ model showed a significant increase in BW and heart weight index (HWI) reaching 135.17 and 115.12%, respectively compared to the normal group. Correspondingly, HFFD/STZ diabetic rats showed significant decrease in HR together with significant prolongation of QTc interval and QRS duration, indicating conduction abnormalities. Although treatment with escitalopram showed no significant decrease in BW, it successfully ameliorated HWI and ECG abnormalities (Table 4).

**TABLE 4:** Effect of escitalopram (10 mg/kg/day; p.o., for 4 weeks) on body weight, heart weight index, and electrocardiographic changes in HFFD/STZ type 2 diabetic rats.

Groups	BW (g)	HWI (mg/g)	Electrocardiographic changes		
			HR (bpm)	QTc (ms)	QRS (ms)
Normal	192.20 ± 5.00	2.91 ± 0.06	339.20 ± 7.33	188.70 ± 6.08	44.50 ± 1.67
Escitalopram	200.00 ± 5.70	2.82 ± 0.07	330.00 ± 6.73	184.50 ± 3.91	43.80 ± 1.60
Diabetes	259.80 ± 12.55*	3.35 ± 0.03*	297.80 ± 5.53*	216.30 ± 4.01*	57.31 ± 1.30*
Diabetes + Escitalopra	242.60 ± 4.55*	3.07 ± 0.08^#^	327.80 ± 5.41^#^	193.00 ± 5.37^#^	49.32 ± 1.31^#^

BW: body weight, HR: heart rate, HWI: heart weight index which is equal to heart weight (mg)/body weight (g). Each value represents the mean of six experiments ±SEM. Statistical analysis was done using ne way ANOVA *followed* by Tukey’s post-hoc test.*p < 0.05 vs. normal, ^#^ *p <* 0.05 vs. diabetes.

### Effect of Escitalopram on Glycemic Profile in HFFD/STZ Type 2 Diabetic Rats

**FIGURE 2:**
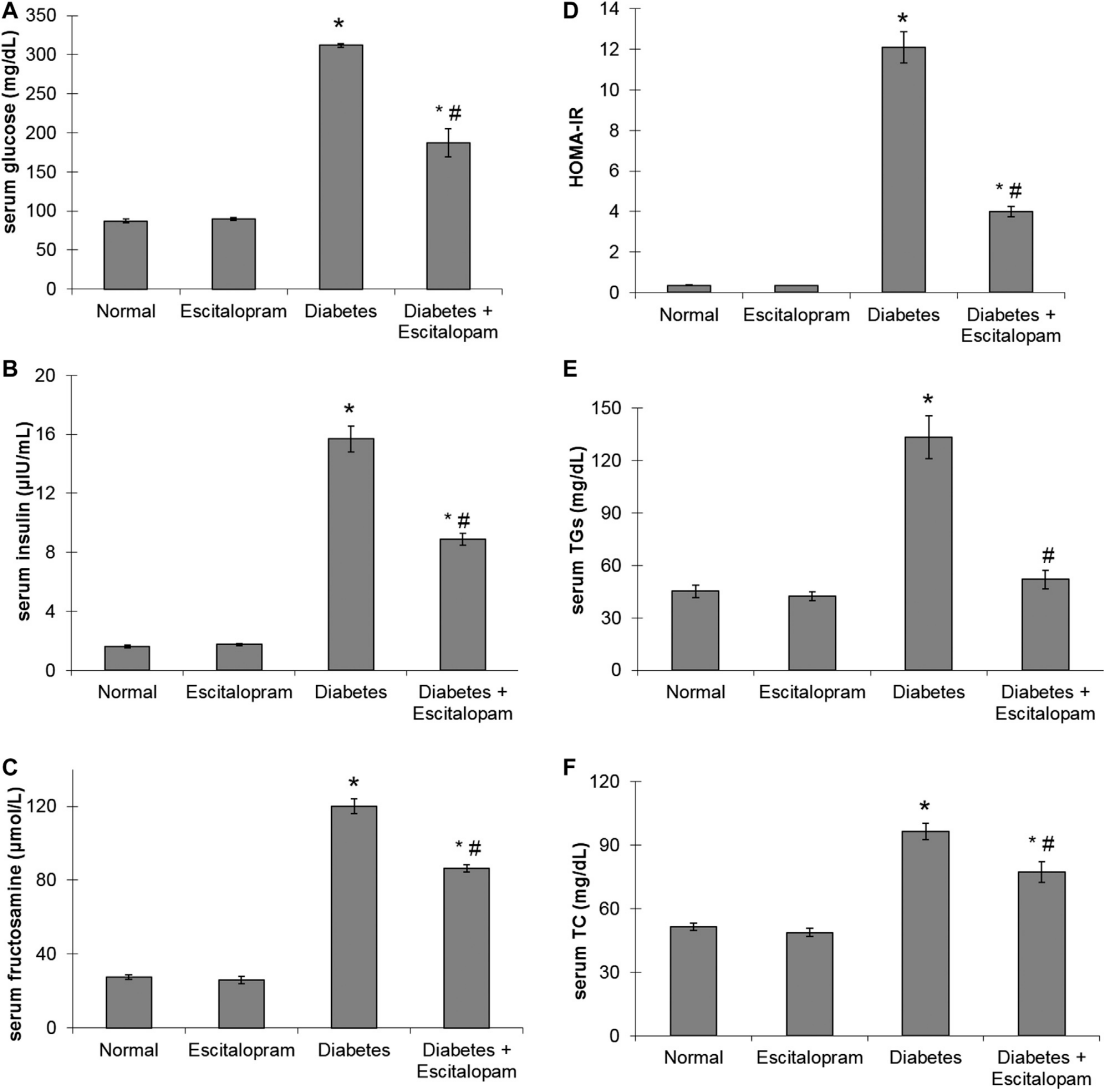
Effect of escitalopram (10 mg/kg/day; p. o., for 4 weeks) on glycemic profile **(A–D)**; serum levels of glucose **(A)**, insulin **(B)**, fructosamine **(C)** and HOMA-IR **(D)** as well as lipid profile **(E, F)**; serum levels of TG **(E)** and TC **(F)** in HFFD/STZ type 2 diabetic rats. Each value represents the mean of six experiments and error bars represent SEM. Statistical analysis was done using One way ANOVA followed by Tukey’s post-hoc test. *p < 0.05 vs. normal, ^#^ *p <* 0.05 vs. diabetes.

A significant increase in serum glucose, insulin and fructosamine levels as well as HOMA-IR by about 258, 863, 335, and 3280%, respectively was noticed in HFFD/STZ diabetic group compared to the normal group. On the other hand, HFFD/STZ diabetic rats treated with escitalopram displayed a remarkable improvement in glycemic control where escitalopram effectively reduced serum levels of glucose, insulin and fructosamine along with HOMA-IR by 40.06, 43.44, 28, and 66.86%, respectively compared to HFFD/STZ diabetic group (Figure 2).

### Effect of Escitalopram on Lipid Profile in HFFD/STZ Type 2 Diabetic Rats

The HFFD/STZ diabetic model was associated with significant increase in serum TGs and TC levels where their values reached 295.59 and 187.86%, respectively compared to the normal group. Escitalopram treatment successfully normalized TGs level and significantly reduced TC level by 19.93% compared to the HFFD/STZ diabetic group (Figure 2).

### Effect of Escitalopram on Myocardial Receptor for Advanced Glycation End Products Content in HFFD/STZ Type 2 Diabetic Rats

**FIGURE 3:**
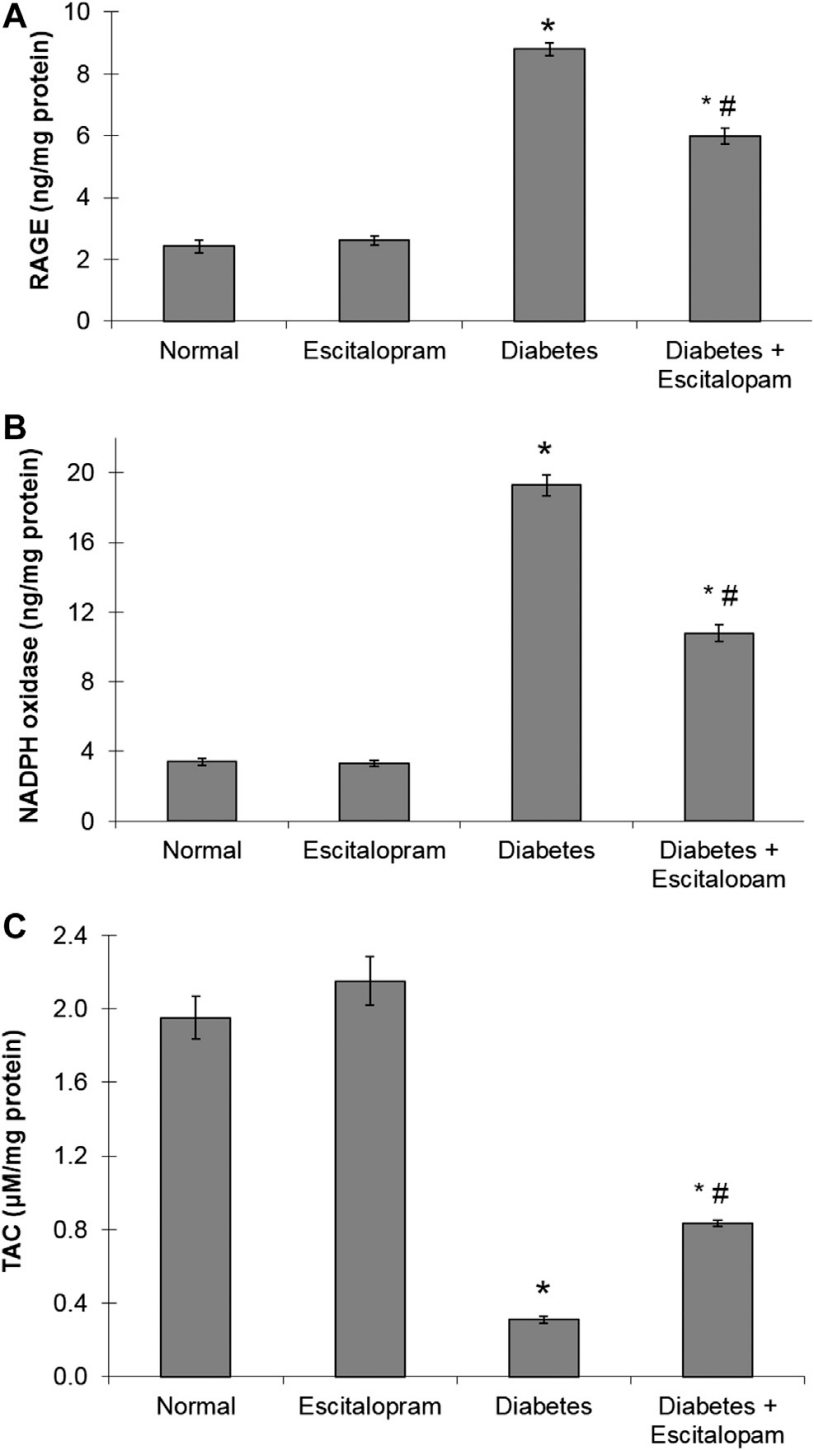
Effect of escitalopram (10 mg/kg/day; p. o., for 4 weeks) on myocardial contents of RAGE **(A)**, NADPH oxidase **(B)** and TAC **(C)** in HFFD/STZ type 2 diabetic rats. Each value represents the mean of six experiments and error bars represent SEM. Statistical analysis was done using One way ANOVA followed by Tukey’s post-hoc test.*p < 0.05 vs. normal, ^#^ *p <* 0.05 vs. diabetes.

The HFFD/STZ diabetic model induced about four fold increase in myocardial RAGE content where this elevation was reversed by escitalopram treatment which significantly reduced RAGE content by 31.93% compared to the HFFD/STZ diabetic group (Figure 3).

### Effect of Escitalopram on Myocardial Oxidative Stress Biomarkers in HFFD/STZ Type 2 Diabetic Rats

The HFFD/STZ diabetic model resulted in a marked increase in the myocardial oxidative stress as indicated by about six fold increase in myocardial NADPH oxidase content along with significant decrease in cardiac TAC to 15.85% compared to the normal group. Escitalopram administration ameliorated the cardiac oxidative stress in HFFD/STZ diabetic rats where it significantly lowered NADPH oxidase content and significantly raised TAC content by 44.04 and 170.23%, respectively compared to the HFFD/STZ diabetic group (Figure 3).

### Effect of Escitalopram on Myocardial Inflammatory and Fibrogenic Biomarkers in HFFD/STZ Type 2 Diabetic Rats

**FIGURE 4:**
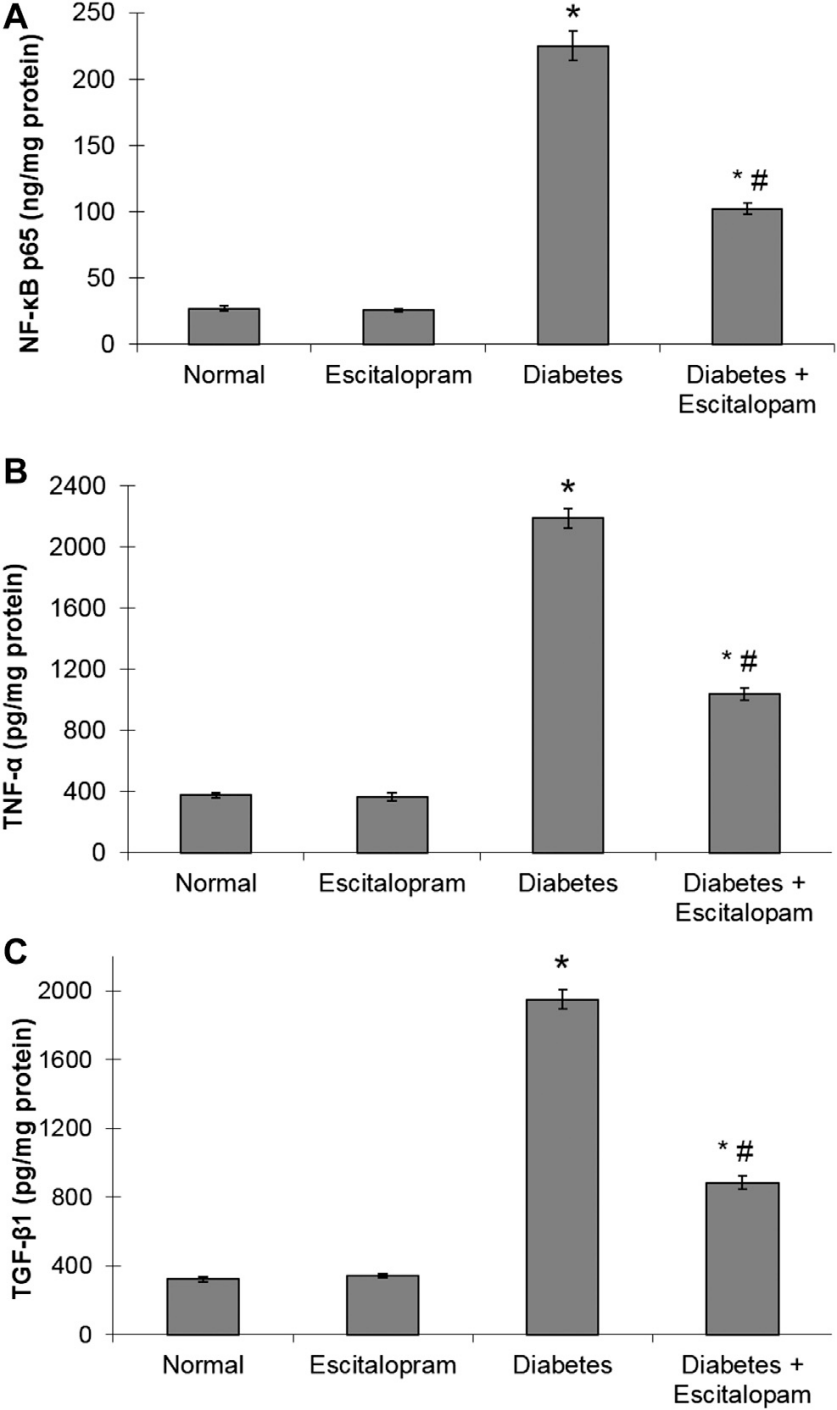
Effect of escitalopram (10 mg/kg/day; p. o., for 4 weeks) on myocardial NF-κB p65 **(A)**, TNF-α **(B)** and TGF-β1 **(C)** contents in HFFD/STZ type 2 diabetic rats. Each value represents the mean of six experiments and error bars represent SEM. Statistical analysis was done using One way ANOVA followed by Tukey’s post-hoc test.*p < 0.05 vs. normal, ^#^ *p* < 0.05 vs. diabetes.

The oxidative stress status was associated with a state of cardiac inflammation and fibrosis that was evident by significant elevation of myocardial NF-κB p65, TNF-α and TGF-β1 contents reaching 833.33, 580.9, and 603.72%, respectively compared to the normal group. Treatment with escitalopram attenuated the HFFD/STZ-induced elevation of inflammatory and fibrogenic markers, where HFFD/STZ diabetic rats treated with escitalopram exhibited a remarkable decrease in NF-κB p65, TNF-α and TGF-β1 contents by 54.67%, 52.51% and 54.72%, respectively compared to the HFFD/STZ diabetic group (Figure 4).

### Effect of Escitalopram on Myocardial Apoptosis in HFFD/STZ Type 2 Diabetic Rats

**FIGURE 5:**
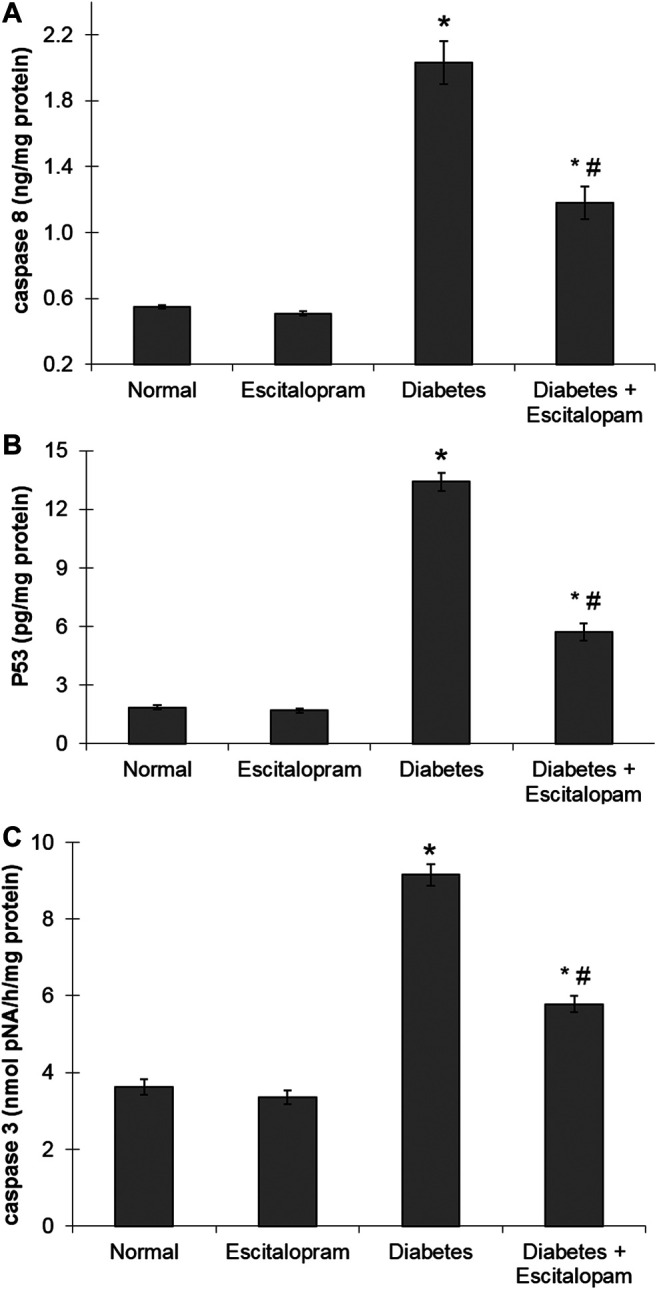
Effect of escitalopram (10 mg/kg/day; p. o., for 4 weeks) on myocardial caspase 8 **(A)** and p53 **(B)** contents as well as caspase-3 activity **(C)** in HFFD/STZ type 2 diabetic rats. Each value represents the mean of six experiments and error bars represent SEM. Statistical analysis was done using One way ANOVA followed by Tukey’s post-hoc test.*p < 0.05 vs. normal, ^#^ *p <* 0.05 vs. diabetes.

The HFFD/STZ diabetic model triggered cardiac apoptosis in rats as evident by the noticeably raised myocardial caspase-8 and p53 contents in addition to caspase-3 activity reaching about four, seven and three fold, respectively. Treatment with escitalopram, on the other hand, mitigated these HFFD/STZ-induced changes decreasing significantly caspase-8 and p53 contents as well as caspase-3 activity by 41.87%, 57.39% and 36.80%, respectively compared to the HFFD/STZ diabetic group (Figure 5).

### Effect of Escitalopram on Myocardial Gene Expression of α-and β-Myosin Heavy Chain as Well as Connexin-43 Protein Expression in HFFD/STZ Type 2 Diabetic Rats

**FIGURE 6:**
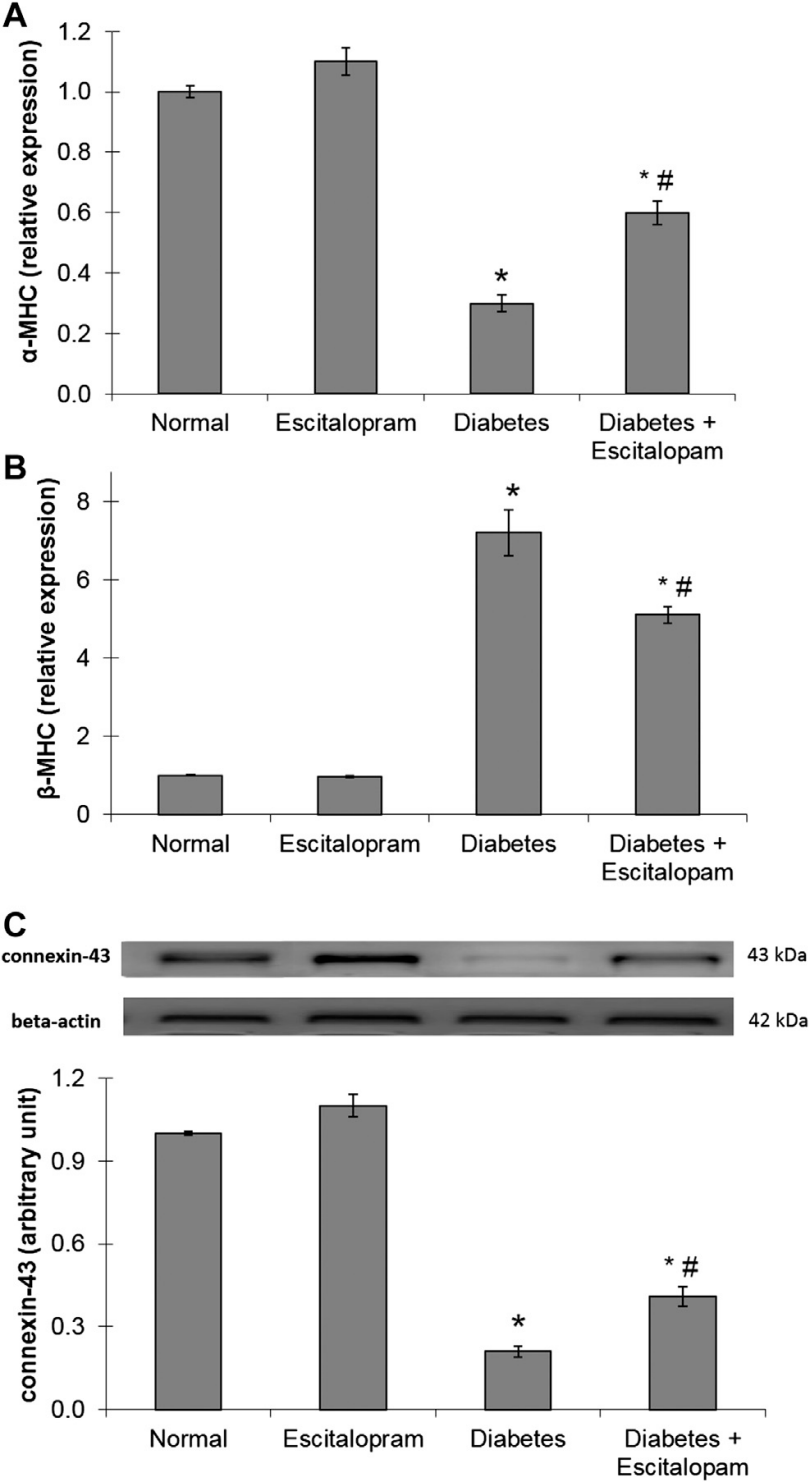
Effect of escitalopram (10 mg/kg/day; p. o., for 4 weeks) on myocardial gene expression of α-MHC **(A)** and β-MHC **(B)** in addition to protein expression of connexin-43 **(C)** in HFFD/STZ type 2 diabetic rats. Each value represents the mean of six experiments and error bars represent SEM. Statistical analysis was done using One way ANOVA followed by Tukey’s post-hoc test.*p < 0.05 vs. normal, ^#^ *p <* 0.05 vs. diabetes.

Myocardial gene expression of α-MHC was significantly decreased in the HFFD/STZ diabetic rats by 70% while β-MHC gene expression was significantly increased by 620%, compared to the normal group. Additionally, the HFFD/STZ diabetic rats displayed a massive decline in the myocardial connexin-43 protein expression indicating impairments of myocardial contractility and conduction. These deleterious changes were antagonized by escitalopram treatment which significantly raised α-MHC gene expression and connexin-43 protein expression while significantly reducing β-MHC gene expression by 100, 95.24, and 29.17%, respectively compared to the untreated HFFD/STZ diabetic group (Figure 6).

### Effect of Escitalopram on Myocardial Histopathology in HFFD/STZ Type 2 Diabetic Rats

**FIGURE 7:**
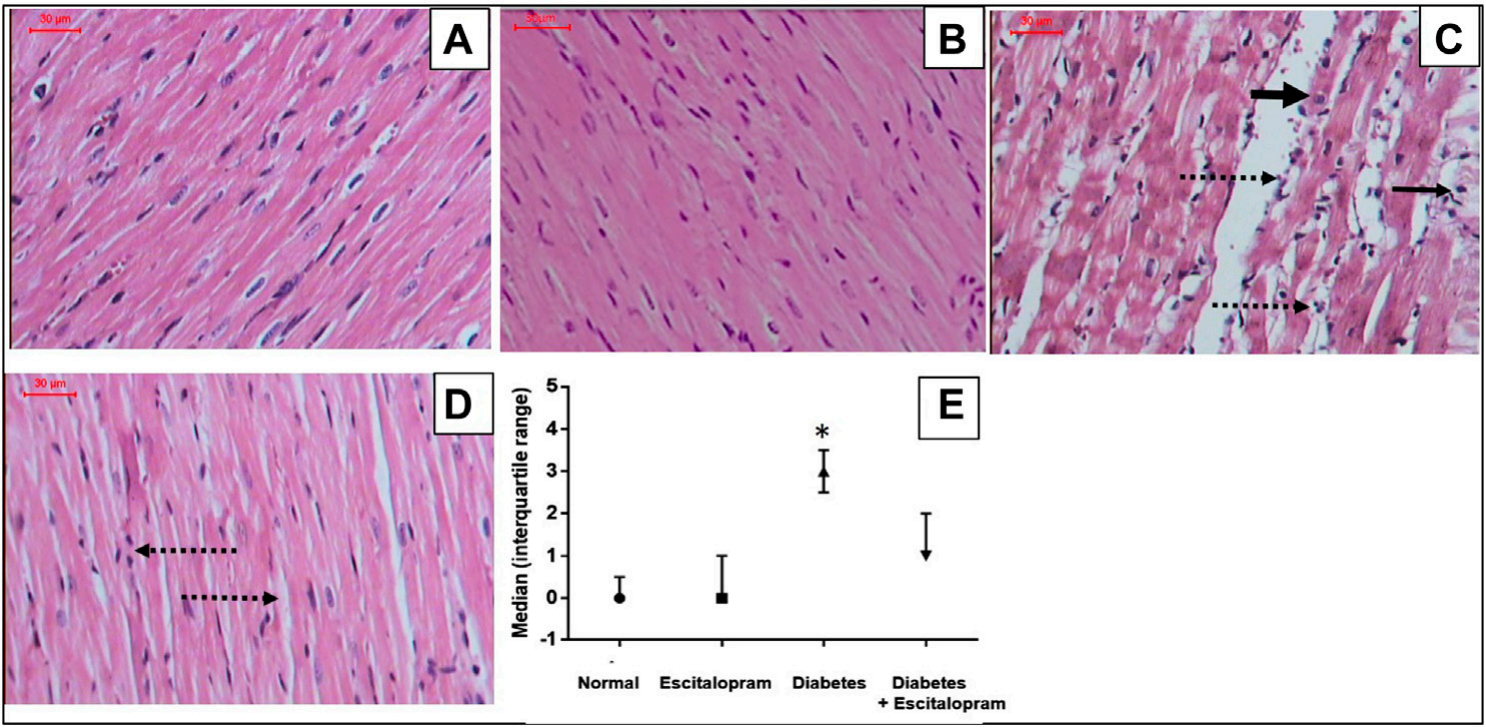
Effect of escitalopram (10 mg/kg/day; p. o., for 4 weeks) on histological damage in HFFD/STZ type 2 diabetic rats. **(A–D)** Specimens stained with H&E (magnification x200). Normal **(A)** and escitalopram **(B)** groups showed normal histological structure of regular cardiomyocytes with normal interstitium. HFFD/STZ diabetes group **(C)** showed showed irregular cardiomyocytes with cytoplasmic vacuolation (narrow arrow), loss of cardiomyocytes striations, focal coagulative necrosis (thick arrow) and marked interstitial edema with inflammatory cellular response (dashed arrow). Diabetes + escitalopram group **(D)** revealed relatively regular cardiomyocytes with mild interstitial edema and inflammatory infiltrate (dashed arrow). Myocardial score of damage expressed as median (interquartile changes) **(E)**. Each value represents the median value [interquartile range]. Statistical analysis was done using non-parametric One-Way ANOVA followed by Dunn's multiple comparison test.*p < 0.05 vs. normal, ^#^ *p <* 0.05 vs. diabetes.

Normal and escitalopram groups showed regular cardiomyocytes with normal interstitium and no evidence of necrosis, vacuolation, interstitial edema or inflammatory response with myocardial score of 0. Meanwhile, the HFFD/STZ diabetes group showed irregular cardiomyocytes with cytoplasmic vacuolation, loss of cardiomyocytes striations, focal coagulative necrosis and marked interstitial edema with inflammatory cellular response recording a myocardial lesion score of 3. On the other hand, treatment of HFFD/STZ diabetic rats with escitalopram ameliorated the HFFD/STZ-induced myocardial changes, showing relatively regular cardiomyocytes with mild interstitial edema and inflammatory infiltrate with myocardial injury score of 1 (Figure 7).

## Discussion

The present investigation was directed to explore the potential beneficial effects of escitalopram on metabolic changes and cardiac complications induced in HFFD/STZ type 2 diabetic rats.

In the current study, the HFFD/STZ rats showed an increment in their BW which could be attributed to the consumption of a diet rich in saturated fats, which would deposit in various body fat pads and decrease energy expenditure (Srinivasan et al., 2005). Furthermore, chronic consumption of diets high in fructose may have deleterious long-term effects on the regulation of energy intake and body adiposity which eventually leads to weight gain, hyperinsulinemia, and the associated IR (Elliott et al., 2002; Basciano et al., 2005).

In the same context, the increase in BW was associated with an altered lipid profile depicted by the elevated serum TGs and TC levels in HFFD/STZ rats. Hypercholesterolemia could be explained by the increased dietary cholesterol absorption from the small intestine following the intake of HFD in a diabetic condition (Colca et al., 1991; Srinivasan et al., 2005) and/or alteration of cholesterol metabolism in diabetic state (Schaalan et al., 2009). Furthermore, fructose can up-regulate the lipogenesis pathway and impair TGs clearance by reducing the activity of lipoprotein lipase as depicted by Thirunavukkarasu et al. (2004). Concerning escitalopram treatment, it caused non-significant reduction in BW of the HFFD/STZ diabetic rats despite ameliorating the HFFD/STZ-induced dyslipidemia. Similarly, escitalopram treatment was reported to ameliorate lipid profile parameters (TC, LDL, TGs) in depressed (Arain et al., 2017) as well as depressed diabetic patients (Singh et al., 2013).

Consistently, in the current study, the HFFD/STZ diabetic rats displayed a significant increase in serum glucose, insulin and fructosamine levels as well as HOMA-IR as previously reported (Schaalan et al., 2009; Elmazar et al., 2013; Amin et al., 2014). Diabetic rats suffered from IR as revealed by the impaired response to the IPGTT and the increase in HOMA-IR index along with the reported compensatory hyperinsulinemia. Increased fatty acid oxidation reduces glucose uptake and utilization in skeletal muscle leading to compensatory hyperinsulinemia, a common feature of IR (Srinivasan et al., 2005). In addition, fructose can induce IR either by decreasing insulin receptor numbers in liver and skeletal muscles or disrupting insulin signaling via reduction of insulin-stimulated receptor autophosphorylation, an essential step for insulin action (Ueno et al., 2000; Catena et al., 2003). The increased fructosamine level in the present study reflects a state of hyperglycemia. Fructosamine is an early glycation end product and can thus be used to predict AGEs concentration (Wilson and Islam, 2015). The present investigation revealed a significant increase in RAGE content where AGEs accumulation up-regulates RAGE (Goldin et al., 2006). Meanwhile, the current data show that escitalopram administration significantly improved the glycemic control in HFFD/STZ diabetic rats. These effects could be explained by the study of An et al. (2009), which showed that escitalopram enhanced net hepatic glucose uptake and hepatic glycogen deposition under hyperinsulinemic hyperglycemic conditions. Furthermore, several studies have highlighted that SSRIs, which increase the level of endogenous serotonin, can improve glucose tolerance and insulin sensitivity in diabetes both experimentally and clinically (Maheux et al., 1997; Gomez et al., 2001).

Dyslipidemia and hyperglycemia induced by HFFD/STZ were accompanied by a state of cardiac oxidative stress as manifested by the elevated myocardial NADPH oxidase content along with the decreased TAC content. AGEs-RAGE interaction generates oxidative stress via the activation of NADPH oxidase (Wautier et al., 2001; Huynh et al., 2014). Excessive production of ROS in diabetes serves to decrease the antioxidant capacity of the diabetic heart, contributing significantly to the resultant myocardial injury (Wold et al., 2005). Hyperglycemia can also impair the endogenous antioxidant defense system (Huynh et al., 2014; Kayama et al., 2015). On the other hand, escitalopram significantly reduced cardiac RAGE content compared to the HFFD/STZ diabetic rats which could be attributed to its beneficial effects on blood glucose and fructosamine levels as well as cardiac oxidative stress biomarkers estimated herein. In the present work, escitalopram conveyed its antioxidant potentials by reducing the NADPH oxidase content and enhancing the TAC in cardiac tissues. The reduced myocardial RAGE content with the expected subsequent decrease in AGEs-RAGE interaction could be responsible for the suppressed NADPH oxidase content as revealed in the current study. The increment in TAC could also be attributed to the decreased consumption of cardiac antioxidant defenses as a result of lowered NADPH oxidase activity and subsequently ROS production.

In addition to oxidative stress, HFFD/STZ diabetic model induced a state of inflammation that was evident by the significant elevation of cardiac NF-κB p65 and TNF-α contents. Cardiac inflammation was also manifested by the marked interstitial edema with inflammatory cellular response observed in the histopathological examination. The activation of NADPH oxidase results in the up-regulation of the oxidative stress-related transcription factor NF-κB and its target genes including pro-inflammatory cytokines such as TNF-α and RAGE itself, thus, creating a positive feedback cycle (Goldin et al., 2006; Lorenzo et al., 2011; Steven et al., 2019) as demonstrated in the present study. Thus, a strong link exists between hyperglycemic-induced oxidative stress, inflammation and the progression of T2DM-induced cardiac complications (Oguntibeju, 2019). TNF-α also contributes to myocardial apoptosis and fibrosis leading to cardiac remodeling and dysfunction (Sun et al., 2007; Frati et al., 2017) and exerting negative inotropic effect on the heart (Frati et al., 2017).

In the present investigation, the pro-oxidant and pro-inflammatory environment in the diabetic heart was associated with marked cardiac fibrosis as well as the activation of both extrinsic and intrinsic apoptotic pathways. The activated NF-κB pathway reported herein has previously been demonstrated to up-regulate the expression of several pro-fibrotic genes such as TGF-β1 and fibronectin, thus promoting the increased extracellular matrix production in diabetic hearts (Aragno et al., 2008; Lorenzo et al., 2011). Indeed, various factors including hyperglycemia, hyperlipidemia, oxidative stress, inflammation and fibrosis have been implicated in inducing myocardial apoptosis in diabetes (Cai and Kang, 2003; Bugger and Abel, 2014). TNF-α is known to induce the extrinsic apoptotic pathway through binding to its receptor resulting in caspase-8 activation, the key initiator caspase of the extrinsic pathway, which in turn activates downstream caspase-3 ultimately leading to cell death (Cai and Kang, 2003). Additionally, p53, a key stimulator of the intrinsic apoptotic pathway, can be activated by hyperglycemia and the excessive oxidative DNA damage in diabetic conditions initiating cardiac cell death (Cai and Kang, 2003; Liu et al., 2009).

Besides its antioxidant effect, escitalopram attenuated HFFD/STZ-induced cardiac inflammation and fibrosis where it significantly reduced NF-κB p65, TNF-α and TGF-β1 contents in cardiac tissues of HFFD/STZ diabetic rats. The present results confirm the anti-inflammatory and anti-fibrotic actions of escitalopram that have been documented in both experimental and clinical studies (Bah et al., 2011; Chavda et al., 2011; van Noort et al., 2014; Ibrahim et al., 2019). The aforementioned antioxidant activity of escitalopram, observed herein, might contribute to the inhibition of NF-κB p65 with the subsequent reduction of TNF-α and TGF-β1 contents.

In the present investigation, the pro-oxidant and pro-inflammatory environment in the diabetic heart was associated with marked cardiac fibrosis as well as the activation of both extrinsic and intrinsic apoptotic pathways. The activated NF-κB pathway reported herein has previously been demonstrated to up-regulate the expression of several pro-fibrotic genes such as TGF-β1 and fibronectin, thus promoting the increased extracellular matrix production in diabetic hearts (Aragno et al., 2008; Lorenzo et al., 2011). Indeed, various factors including hyperglycemia, hyperlipidemia, oxidative stress, inflammation and fibrosis have been implicated in inducing myocardial apoptosis in diabetes (Cai and Kang, 2003; Bugger and Abel, 2014). TNF-α is known to induce the extrinsic apoptotic pathway through binding to its receptor resulting in caspase-8 activation, the key initiator caspase of the extrinsic pathway, which in turn activates downstream caspase-3 ultimately leading to cell death (Cai and Kang, 2003). Additionally, p53, a key stimulator of the intrinsic apoptotic pathway, can be activated by hyperglycemia and the excessive oxidative DNA damage in diabetic conditions initiating cardiac cell death (Cai and Kang, 2003; Liu et al., 2009).Expectedly, escitalopram treatment managed to ameliorate diabetes-induced cardiac apoptosis as evidenced by decreased cardiac caspase-8 and p53 contents as well as caspase-3 activity. The reduced caspase-8 content could stem from the decline in TNF-α which upon binding to its receptor causes caspase-8 activation (Cai and Kang, 2003). While, the reported anti-hyperglycemic and antioxidant effects of escitalopram, in the present study, could be responsible for the decreased p53 content as hyperglycemia-induced oxidative DNA damage is a known stimulator for p53 upregulation (Cai and Kang, 2003; Liu et al., 2009).

The previously mentioned biochemical alterations in HFFD/STZ diabetic rats were correlated with significant increase in HWI along with a switch from α-to β-MHC gene expression and reduced connexin-43 protein expression indicating hypertrophy and conduction impairments in the present study. This was confirmed by electrocardiographic alterations as revealed by significant decrease in HR together with significant prolongation of QTc interval and QRS duration. The remarkable cardiac hypertrophy along with the shift from α-to β-MHC gene expression have been reported in fructose fed type 2 diabetic rats (Bagul et al., 2015) which could be partially responsible for the reduced contractile function in diabetic hearts (Shao et al., 2010). Moreover, connexin-43 is vital for normal cardiac excitation and contraction, and the decrease in its expression may account for impaired impulse propagation and conduction abnormalities in diabetic hearts (Lin et al., 2006; Grisanti, 2018) as reported herein. Notably, the present data reveal that escitalopram improved the HFFD/STZ-induced cardiac hypertrophy as well as contraction and conduction impairments as evidenced by the normalization of HWI, HR, QTc interval and QRS duration in addition to significant amelioration of α- and β-MHC genes expression and connexin-43 protein expression. The modulatory effects of escitalopram on RAGE content and the consequent reduction of oxidative stress, inflammatory and fibrogenic markers might be responsible for preventing the observed disruption of the aforementioned parameters. Further studies are required to clarify in detail the exact mechanism involved in the beneficial effect of escitalopram on diabetic cardiomyopathy.

In summary, the current study demonstrated that escitalopram alleviated the HFFD/STZ-induced metabolic derangements as revealed by improvement of both lipid and glycemic profiles. Furthermore, escitalopram treatment showed beneficial effects towards reducing the HFFD/STZ-induced cardiac oxidative stress, inflammation, fibrosis as well as conduction and contraction impairments which could be related to its observed beneficial effects on glycemic control and RAGE content. Thus, escitalopram could be considered a favorable antidepressant medication in diabetic patients as it seems to positively impact the glycemic control in diabetes in addition to prevention of its associated cardiovascular complications.

## Data Availability Statement

The raw data supporting the conclusions of this article will be made available by the authors, without undue reservation.

## Ethics Statement

The animal study was reviewed and approved by Ethics Committee, Faculty of Pharmacy, Cairo University (Permit Number: PT 1303).

## Author Contributions

LAA, NAS and ASA developed the idea and designed the experimental approach. LAA and NAS performed the experiments, analysed the data, wrote the main manuscript text and prepared the tables and figures. All authors reviewed and approved the final manuscript.

## Funding

This research received no specific grant from any funding agency in the public or commercial.

## Conflict of Interest

The authors declare that the research was conducted in the absence of any commercial or financial relationships that could be construed as a potential conflict of interest.
